# Supplementary figures and images for: Meteorological factors and non-pharmaceutical interventions explain local differences in the spread of SARS-CoV-2 in Austria
Source: PLoS Comput Biol. 2022 Apr 4;18(4):e1009973. doi: 10.1371/journal.pcbi.1009973 (PMC9009775; doi:10.1371/journal.pcbi.1009973)

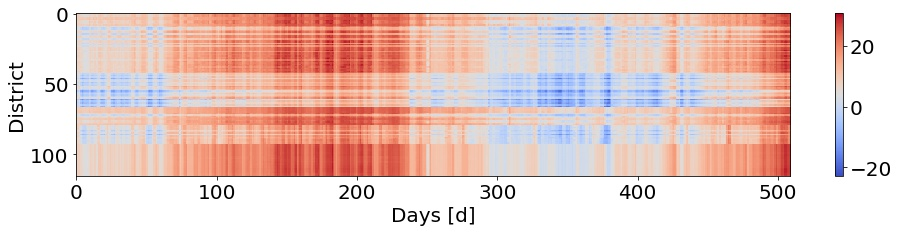

Supplement: S1 Fig — (TIF) [file pcbi.1009973.s001.tif]

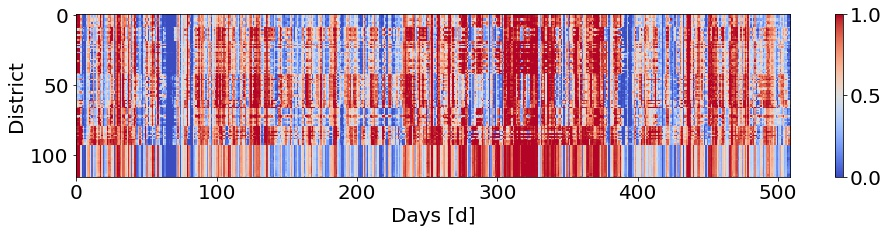

Supplement: S2 Fig — (TIF) [file pcbi.1009973.s002.tif]

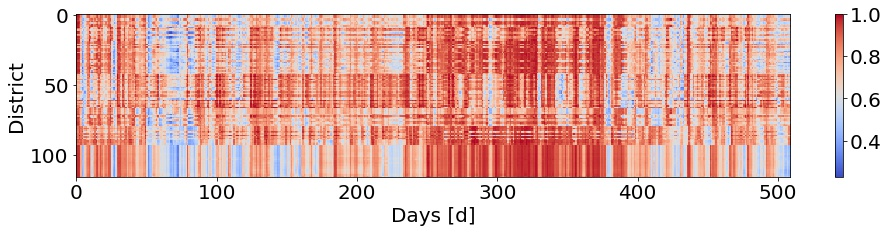

Supplement: S3 Fig — (TIF) [file pcbi.1009973.s003.tif]

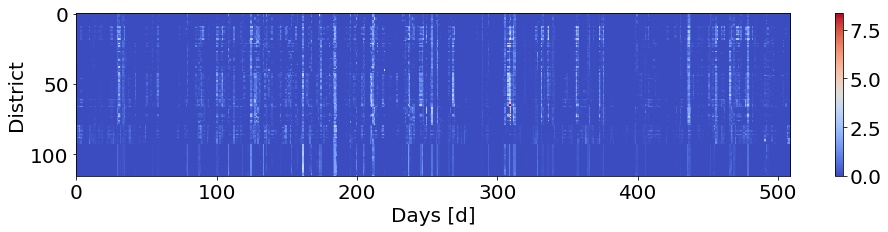

Supplement: S4 Fig — (TIF) [file pcbi.1009973.s004.tif]

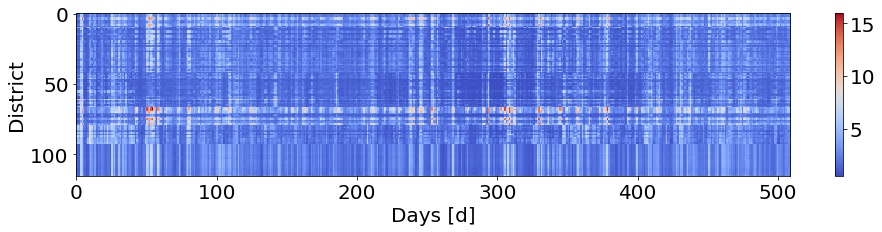

Supplement: S5 Fig — (TIF) [file pcbi.1009973.s005.tif]

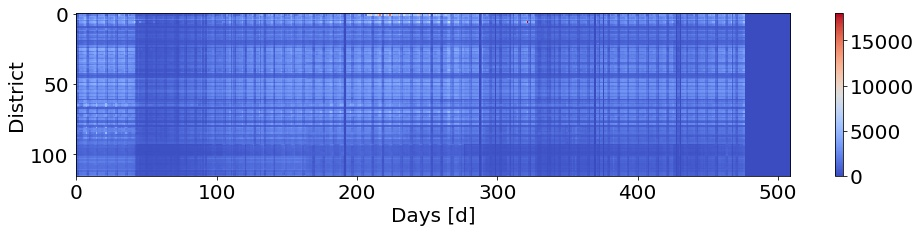

Supplement: S6 Fig — (TIF) [file pcbi.1009973.s006.tif]

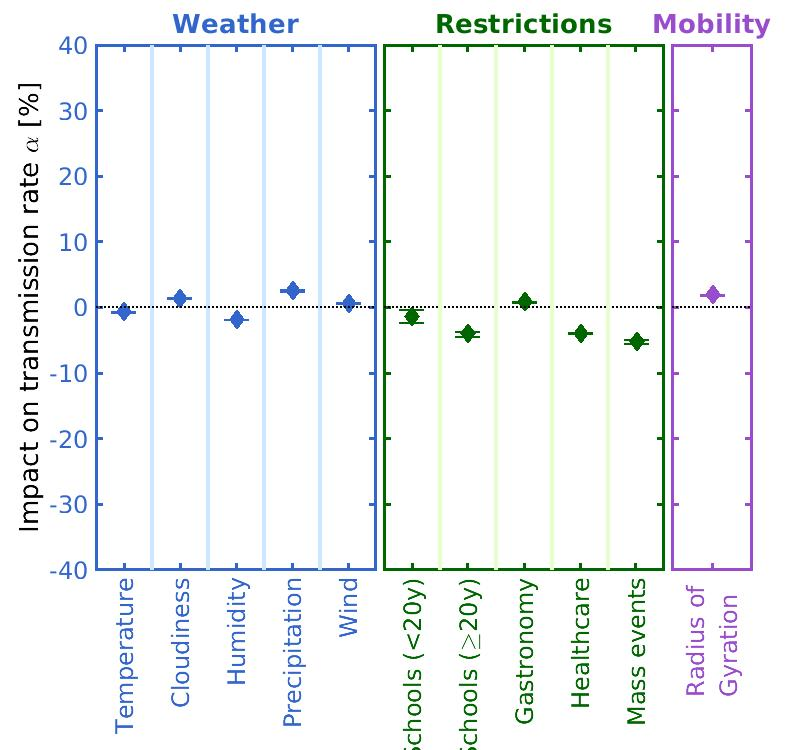

Supplement: S7 Fig — (TIF) [file pcbi.1009973.s007.tif]

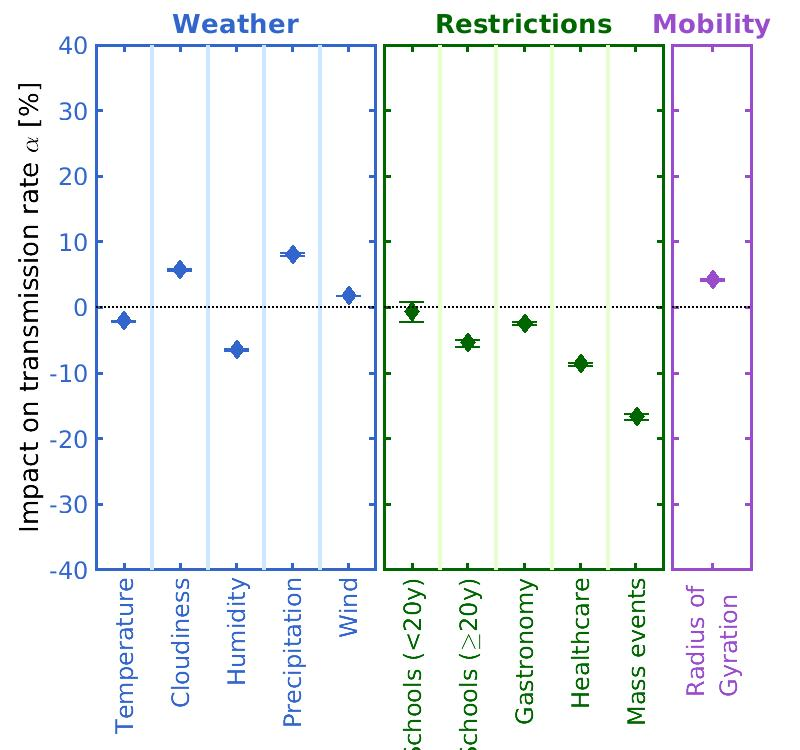

Supplement: S8 Fig — (TIF) [file pcbi.1009973.s008.tif]

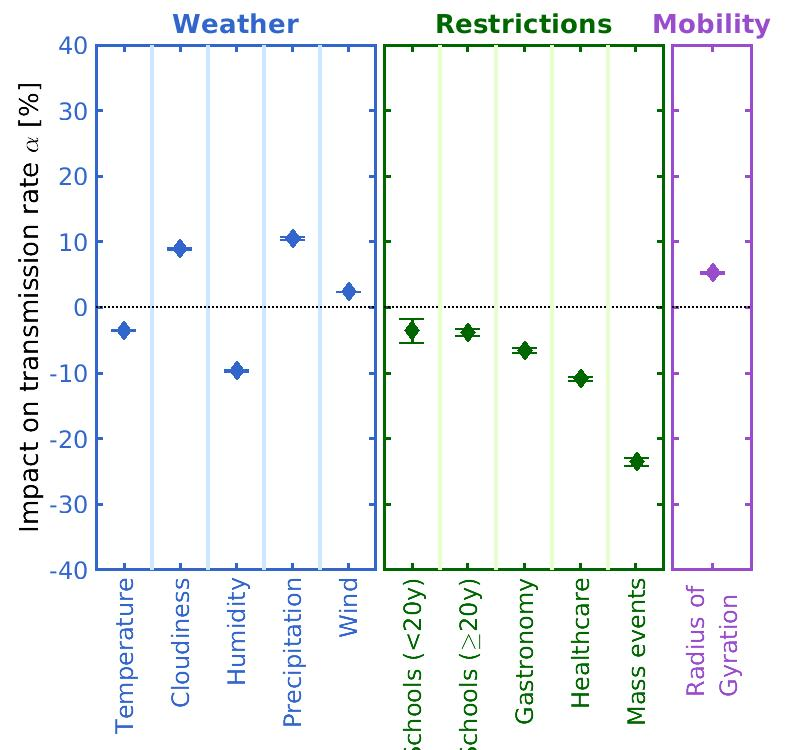

Supplement: S9 Fig — (TIF) [file pcbi.1009973.s009.tif]

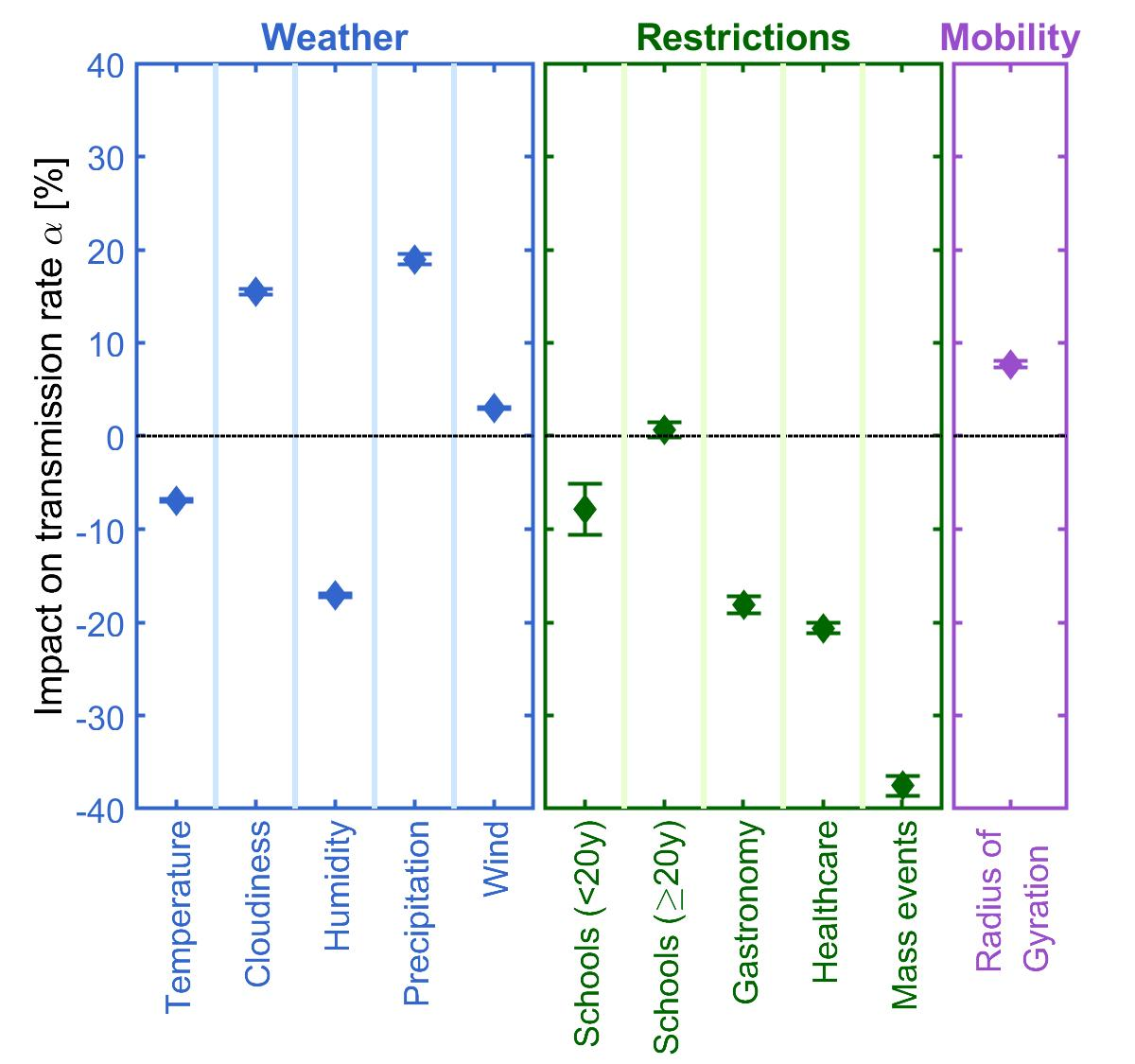

Supplement: S10 Fig — (TIF) [file pcbi.1009973.s010.tif]

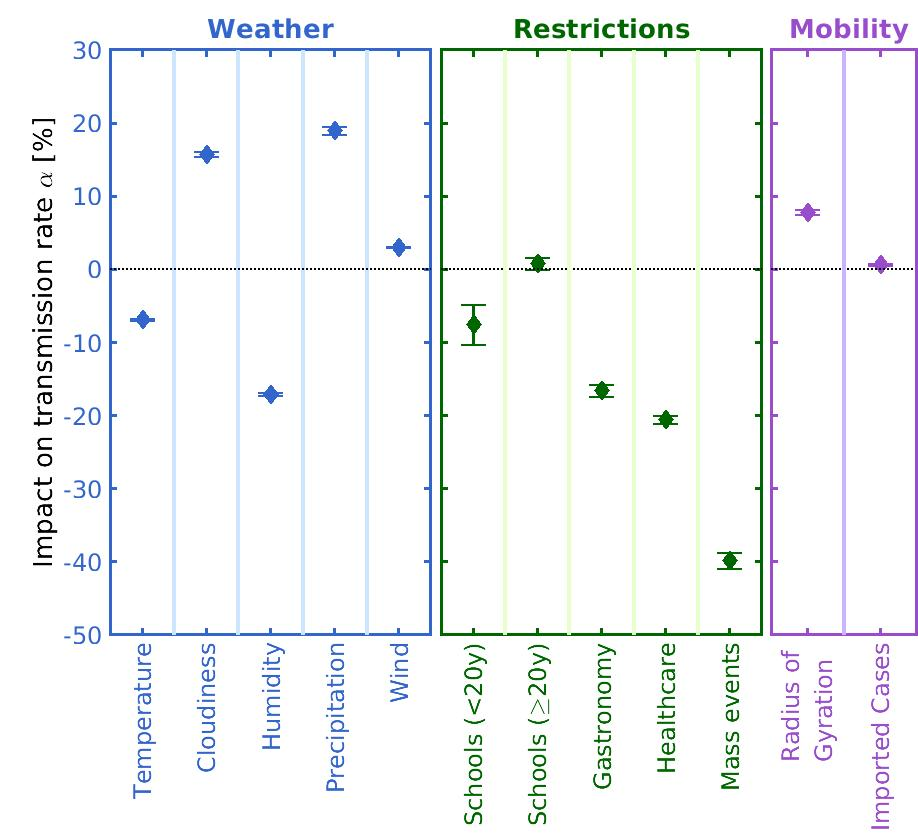

Supplement: S11 Fig — (TIF) [file pcbi.1009973.s011.tif]
