# Supplementary material for: Meteorological factors and non-pharmaceutical interventions explain local differences in the spread of SARS-CoV-2 in Austria
Source: PLoS Comput Biol. 2022 Apr 4;18(4):e1009973. doi: 10.1371/journal.pcbi.1009973 (PMC9009775; doi:10.1371/journal.pcbi.1009973)
Supplement: S1 Table — Table showing for each restriction how many days the restriction was active and in how many different districts it occurred. (PDF) [file pcbi.1009973.s012.pdf]

	School	Gastronomy	Healthcare	Mass events
Total days active	21034.0	10191.0	11249.0	5189.0
# Districts	108.0	87.0	98.0	63.0

Table S 1: This table shows for each restriction how many days the restriction was active and in how many different districts it occurred.
