# Supplementary material for: Meteorological factors and non-pharmaceutical interventions explain local differences in the spread of SARS-CoV-2 in Austria
Source: PLoS Comput Biol. 2022 Apr 4;18(4):e1009973. doi: 10.1371/journal.pcbi.1009973 (PMC9009775; doi:10.1371/journal.pcbi.1009973)
Supplement: Attachment — Submitted filename: Reviewer comments .pdf [file pcbi.1009973.s013.pdf]

We thank the referees for their time and diligence that went into reviewing our paper. The referees have identified a number of issues that needed to be clarified, thereby further improving the quality of our manuscript, for which we want to express our deep gratitude. We have taken each of their comments thoroughly into account and changed the manuscript accordingly. In particular, we have made substantial revisions by changing the treatment of the hyperparameters to obtain more meaningful confidence intervals and by adding additional robustness tests. For a detailed point-by-point response, see below.

Reviewer #1:

The authors developed an SIR model with the main goal of understanding fine spatio-temporal differences of SARS-CoV-2 transmission in the 116 districts of Austria. The authors put emphasis on meteorological factors, including wind, temperature, etc. Moreover, age-mixing and non-pharmaceutical interventions (NPI) are considered to understand differences in transmission in the districts. The model combines an impressive number of different data sources, including meteorological data, mobility data, NPI and SARS-CoV-2 cases. The model is well described and the manuscript is interesting to read.

> *We thank the reviewer for providing us with very helpful remarks and are glad to see that the manuscript was interesting to read.*

However, as with every mathematical model, parameter choices and assumptions need to be made to perform such a study. Although the authors include a variety of different factors, of course, not all factors influencing SARS-CoV-2 transmission can be included, and some simplifications need to be made. The authors put much emphasis on the percentage of transmission variation that can be explained by meteorological factors and NPI: however, given the model simplifications and parameter choices, I am not sure whether this number (i.e. 60%) is something one should put emphasis on. To me, it is not clear, what we can really learn from the results presented here. If 60% of the variation can be explained by the factors included in the model, what about the remaining 40%? What would happen if additional factors would be included (e.g. nation-wide NPIs, etc.?).

> *In the revised version we have added additional robustness tests to put these numbers in perspective. In particular, we show how the proportion of the explained variance changes if particular sets of variables are excluded from the analysis. This should allow readers to better understand how much of the observed regional patterns of spread can be explained by weather vs NPIs vs mobility. For the remaining 40%, however, we can only speculate what might cause these variations. Potential reasons include socio-economic or demographic differences in the populations or limitations that arise due to inaccuracies in determining the model input variables. Finally, to which extent more or less unpredictable singular super-spreading events might contribute to the observed variation is also not clear.*

One suggestion would be to fit the model again excluding meteorological factors and compare the results with the original model: with this, one would get an estimate how much of the variation can be explained by meteorological factors. The same strategy for the other factors.

> *We thank the reviewer for this suggestion. We included a section in the manuscript in order to investigate the robustness of the model. There we show the decrease in explained variation when a variable group (meteorological factors, NPIs and mobility) is excluded. We included the decrease in explained variation once for re-fitting the model and once for re-running the model. We found that the NPIs is the variable group which is the major contributor to the explained variation. The new results are now described in the result section "robustness".*

Major comments/questions concerning parameter choices and model design:

1)Age-mixing: To me, it is not clear how age-mixing was determined. For whole Austria, the authors seem to use the matrix presented in Prem et al. However, the study by Prem et al was done before the Corona pandemic, and hence, mixing might be different from before: especially given "home office", bans of large events, home schooling, etc., I would assume that mixing in 2020 was different compared

to mixing <2017. Moreover, I do not understand ow age- mixing was calculated for the districts. More on a general note: Why is so much emphasis on age-mixing, is this really necessary for the study question of interest? I would completely drop this stratification.

Thank you for making us aware of this limitation concerning the age-dependent mixing; we now explicitly discuss this limitation in the discussion section. The main motivation for including age-dependent mixing was that we considered NPIs – restrictions in schools – that target specific age groups. Our study therefore allows us to disentangle effects of such restrictions in school-aged cohorts from effects observed in adults. This partially also answers the concern regarding measures that might change age-dependent contact patterns – this is exactly what we seek to capture with this stratification. As discussed in methods, we calculate the social mixing matrices by assuming that the contact probability for someone of age a to meet a' is the same in each district and then weight these probabilities according to the number of people in each age group specifically in the districts.

2)Recovery rate: I am confused about the interpretation of this parameter. Looking at the model, beta is the rate at which individuals move from the infected department to the recovered department. Individuals in the infected department are able to infect susceptible individuals. Hence, the recovery rate is describing the number of days an infected individual is able to infect a susceptible individual. The authors fix this rate at 25 days, which is – given the isolation and quarantine for infected individuals, much too long. It might take 25 days on average to recover from Covid-19 disease, but the number of days an individual can infect others should be much lower. In line 303-305, this authors state that the recovery rate should be interpreted as the time span in which changes in NPIs or weather events might influence transmission rate: I do not see why this would be an appropriate interpretation. Looking at the model, beta clearly is the time an individual can infect a susceptible individual.

Als in response to the other cases, we substantially changed how we deal with the recovery rate. In particular we consider the recovery time now as being drawn from a distribution, fix the value of the recovery time, compute the model effects, and then take the ensemble average over all models with a weight given by the probability to observe the recovery rate with a given probability. This way we can reduce a potential problem of overfitting with a fixed recovery time and also alters the interpretation of our results. We have therefore removed this discussion as suggested by the referee and rather discuss now a motivation for handling the recovery rate as we do now.

3)The authors fit the model to the number of confirmed SARS-CoV-2 cases. Ideally, these data points should be the most reliable ones. However, there were different testing strategies in Austria in these 10 months of interest. If possible, the authors should include estimates on total number of tests or negative tests as well to get a more reliable number concerning total cases. Differences in testing might also explain why the model explained the two waves not with the same precision.

For exactly this reason, the choice of the control set in our work was also motivated by the fact that in Austria each federal state has its own health authority that is responsible for, e.g., the testing strategy and contact tracing. Hence, our analysis controls for differences in testing and tracing. We state this now explicitly when describing the construction of the control set, that this definition ensures that we always compare (sets of) districts that had the same test and trace strategy in place.

4)The model does not capture infections from outside the district. This feels like a simplification, which could have a huge impact – especially for districts of the larger cities. I am wondering whether one could add an additional parameter in the infected equation to reflect additional infections, like: I + lambda*S – beta*I + gamma to model a time-varying transmission from "outside".

As an additional robustness test we did include a time series capturing the imported cases (based on mobility data), as a variable. That is, we computed an indicator that gives an estimate for the number of people that entered a given district from another one, weighted by

the current incidence in the source district. However, in the model adding this indicator did not show a significant effect size on the transmission rate and we chose to exclude it. However, the reviewer is correct, it is an important finding and we now include a summary of effect sizes including the imported cases in the SI. Our interpretation of this finding is that most of the imported cases can be expected to come from districts that are members of the control set and therefore there is little additional explanatory effect coming from adjusting for the incidence level in surrounding areas also using mobility data.

5)Home schooling: I am confused by the results concerning home schooling. In Line 159, the authors mention that an effect for home schooling was only considered for age < 20y. Later on, 197-199, the authors state as one of their main results that home schooling only had an effect for age < 20y. Isn't this result a consequence of the model design? I do not understand this point, and Line 265-268 introduce even more confusion concerning interpretation of these home schooling results.

An effect of the NPI restrictions in schools (i.e singing in classrooms, cloth masks when not seated etc.) was considered both for the population < 20 y and > 20 y. However, the effect was calculated individually for age below or above 20 y in order to be able to make separate conclusions of the efficacy on the infection process in the age groups respectively. Since we found that there is no significant effect of restrictions in schools, one could assume that this NPI does not affect the population above 20 y because the infection dynamics in schools are decoupled from the ones outside. In line 265-268 we want to make sure that this is not the reason for the non-significance of this effect. The reason is that the effect of school restrictions on infectious dynamics among the population older than 20 y, is embedded in the structure of the social mixing matrix (less infected children infect less adults).

6)I am missing a discussion about district size: Is the city of Vienna one district? Is the population of Austria evenly distributed in the districts? As the federal state is the reference for the calculations, it would be interesting to know how the districts are distributed in the federal states. In general, I am wondering whether federal state as a reference is the best choice: One could, for example, take as a reference all neighboring districts independent of the federal state – especially since weather (as opposed to NPIs) should be similar in neighboring districts.

As we describe now in the manuscript and have already discussed above, the choice of the control set was mainly motivated by the fact that federal states govern the test-trace-isolate strategy via their regional authorities and therefore comparability within a federal state is larger than across federal states. Furthermore, we have added a description of district sizes and how we handled the city of Vienna (namely as separated into its 23 districts).

7)Given all the uncertainties that come with the model, I am really surprised to see very narrow confidence intervals in Figure 3. How were these intervals computed? And what is the authors take on "over-fitting"?

Thank you for bringing this issue to our attention. These confidence intervals are indeed results from the fitting when fixing beta. In the revised version we accommodate this issue by also varying beta as a hyperparameter and by reporting effect sizes as ensemble average over all plausible hyperparameter settings. Consequently, confidence intervals are now also computed via standard deviations associated with this ensemble average, resulting in larger confidence intervals.

Reviewer #2:

PCOMPBIOL-D-21-01637

Meteorological factors and non-pharmaceutical interventions explain local differences in the spread of SARS-CoV-2 in Austria

Summary and comments to the authors

The above-mentioned manuscript develops a modelling approach to quantify the effect of meteorological variables and non-pharmaceutical interventions in the spread of SARS-CoV-2 in Austria. I think the paper is interesting and well written but I have the following comments:

- Figure 1 should change a bit to help readers understand the spatial context of Austria. Namely, I suggest to change the background blue of the states to white or something else, so it is clear that the blue is only Tyrol. You need to add some explanation with respect to what the larger blue regions in Tyrol represent and how they differ from districts. Can you add a map of the district boundaries with some statistics, such as population and area as a supplement?

> *In Figure 1 we changed the color of the background from blue to gray for better visibility. We additionally explain now in the figure description that the larger blue regions in Tyrol do represent the districts of Tyrol and refer to the website of Statistics Austria where the districts are listed and statistics such as the population per district can be found.*

- I would also add on a supplement some maps of the meteorological covariates, say mean over the time period, so the reader can see the extend of spatial misalignment (outcome-covariate) existing in the data.

> *In the SI heat maps of the different time series of the weather data across all districts are added as well as tables with some statistics on the NPIs.*

- You mention that you use only confirmed COVID-19 cases in the model. Are these confirmed with a PCR or LFT? Does it makes sense to account for the sensitivity and specificity of these tests? How good the testing coverage is in Austria and how many cases do you expect to miss? Is this missing at random or can it introduce biases to your estimates of meteorology?

> *We use case data from the official epidemiological reporting system which ensures that a consistent case definition has been applied across all districts (that vast majority of cases being PCR confirmed, there were short spells of time in which for symptomatic contact persons also LFT-positivity was accepted in the case definition if administered from health personnel). We acknowledge as a limitation that our parsimonious modeling approach gives no representation to undetected cases. However, as we describe now in Methods when motivating our choice for the control set, federal states in Austria have their own health authorities that are in charge of implementing test-trace-isolate. This means that as we only compare districts with a control set consisting of districts from the same federal state, our analysis controls for potential differences in testing and contact tracing, which should considerably alleviate biases that might arise from differences in the test strategy.*

- I was wondering if you could show on a supplement how the effect estimates of the meteorological covariates or NPI change with the different \beta. You could select the extremes and show the variation, or you could even fit all selected \beta and create an ensemble estimate of the covariates, by combining the results, whatever you think makes more sense in this particular context.

> *Thank you for this suggestion to revisit this issue. We included a section where we check the robustness of the model. There we include an ensemble estimate of the covariates for different \beta ranging from 14d to 42d. We calculate a weighted average of the resulting effect sizes using a gamma distribution from Estimation of COVID-19 recovery and decease periods in Canada using delay model, Sci Rep 11, 23763 (2021) with a maximum at 19d.*

- You mention briefly in the discussion that the meteorological factors might be confounded with behavioural aspects. This behavioural aspects that vary by country, might explain the inconsistent results of the literature. I think this merits more discussion.

We have expanded this discussion. However, in another round of literature search we were not able to locate new evidence that seeks to disentangle physical from behavioural weather effects. We observe that our mobility indicators should partially allow to isolate beavioural effects such as staying at home. However, we note that our findings here are rather limited and further research is needed in order to disentangle these dimensions.

- A Bayesian approach, would have resulted more natural uncertainty results, and also will have helped propagating any uncertainty that comes with the selection of \beta.

We have substantially changed the approach to incorporating uncertainties associated with \beta substantially. In brief, we now consider an ensemble of models with their own \betas. The weights for the ensemble members are drawn from a distribution that corresponds to the observed distribution of infectiousness durations. Our resulting effect estimates and their uncertainties are then computed over the ensemble of models.

- I would assume that the data is open available, and thus suggest the code and the data to be put on a repository, to help reproducibity of the study.

The availability of the data is, partly, yet to be determined by the providers. However, as soon as this is cleared the data will be provided in a repository upon completion.

Reviewer #3:

In "Meteorological factors and non-pharmaceutical interventions explain local differences in the spread of SARS-CoV-2 in Austria" authors study a critically relevant and timely problem, and they provide innovative insights that help us better understand and manage the COVID-19 pandemic in terms of a rigorous and data supported approach.

How to best contain and control the spread of COVID-19 is of outstanding and immediate importance. I have enjoyed reading this paper. I find it comprehensive and clearly written, and introducing new results that will surely inspire future research along similar lines. The main messages are brought across fully supported by the presented results, and the writing is reasonably accessible to the wider audience. For these reasons, I am in favor of revisions for PLOS Computational Biology as follows.

1) In the introduction, I am missing a couple of references where similar regional data has been studied for other countries in Europe. Perhaps not necessarily with the focus on meteorological factors, but nevertheless closely related. For a topical piece for the whole of Europe, the following reference also seems very much fitting: Towards a European strategy to address the COVID-19 pandemic, Lancet 398, 838-839 (2021).

We thank the reviewer for the literature suggestion. We included the suggested reference and added relevant, similar literature in the introduction.

2) A note on the robustness of the experimental data used for parameter estimation would be welcome given the still relatively varying rates available in the literature for various countries and regions of the world. A note on limitations that may not be apparent to readers as to why no robustness check were made would also be useful.

We have performed a number of additional robustness tests that are now described in the results section. In brief, these robustness tests include additional variables for imported cases and various types of drop out experiments (re-running the models for excluded sets of parameters or re-fitting the model to subsets of variables).

3) I also do not agree so much with the statement that less attention has been paid to more fine-scaled spatio-temporal variations in virus spread. This has in fact been studied recently also in Community lockdowns in social networks hardly mitigate epidemic spreading, New J. Phys. 23, 043039 (2021) and Socio-demographic and health factors drive the epidemic progression and should guide vaccination strategies for best COVID-19 containment, Results Phys. 26, 104433 (2021).

We thank the reviewer for making us aware of the two references on spatio-temporal variations in virus spread. We included these articles in our manuscript.
